# Efficacy and safety of follitropin delta versus follitropin alpha/beta in infertility treatment: A systematic review and meta‐analysis

**DOI:** 10.1002/rmb2.12573

**Published:** 2024-03-25

**Authors:** Shinnosuke Komiya, Jun Watanabe, Takero Terayama, Kyosuke Kamijo, Hidetaka Okada

**Affiliations:** ^1^ HORAC Grand Front Osaka Clinic Osaka Japan; ^2^ Department of Obstetrics and Gynecology Kansai Medical University Graduate School of Medicine Hirakata, Osaka Japan; ^3^ Systematic Review Workshop Peer Support Group (SRWS‐PSG) Osaka Japan; ^4^ Division of Gastroenterological, General and Transplant Surgery, Department of Surgery Jichi Medical University Shimotsuke Japan; ^5^ Division of Community and Family Medicine Jichi Medical University Shimotsuke Japan; ^6^ Division of Traumatology and Critical Care Medicine National Defense Medical College Saitama Japan; ^7^ Division of Gynecology Nagano Municipal Hospital Nagano Japan

**Keywords:** assisted reproductive techniques, female infertility, fertilization in vitro, follicle‐stimulating hormone, ovarian hyperstimulation syndrome

## Abstract

**Background:**

Follitropin δ may be an alternative to conventional follitropin α/β for controlled ovarian stimulation (COS) within assisted reproductive treatment (ART), but its efficacy and safety remain unknown. We performed a random‐effects meta‐analysis to compare the efficacy and safety of follitropin δ and follitropin α/β.

**Methods:**

We searched randomized controlled trials comparing follitropin δ and follitropin α/β using MEDLINE, Embase, CENTRAL, ClinicalTrials.gov, and WHO‐ITCRP on December 14, 2022. The primary outcomes were the live birth rate and the incidence of moderate or severe ovarian hyperstimulation syndrome (OHSS). The certainty of the evidence was assessed using the grading of recommendations assessment, development, and evaluation approach. The protocol was registered on the Open Science Framework.

**Results:**

Three studies involving 2682 participants were included in our meta‐analysis. The results indicated that follitropin δ may result in little to no difference in live birth rates (risk ratio [RR], 1.12; 95% confidence interval [CI], 0.91–1.38; low certainty) and the incidence of moderate or severe OHSS (RR, 0.78; 95% CI, 0.48–1.26; low certainty) compared with follitropin α/β.

**Conclusion:**

Follitropin δ may result in little to no difference in COS compared with follitropin α/β, especially in terms of live births and safety.

## 1 INTRODUCTION

Optimizing fertility outcomes without sacrificing safety is a crucial goal in reproductive medicine. Several follicle stimulation protocols regulated by gonadotropins have been developed, with various mechanisms for suppressing ovulation.^1^, ^2^, ^3^ The antagonist protocol using gonadotropin‐releasing hormone (GnRH) antagonists has attracted attention for its ability to minimize the risk of ovarian hyperstimulation syndrome (OHSS), as a potentially severe complication.^4^, ^5^ Follitropin α and β are commonly used recombinant gonadotropins whose efficacies have been increasingly examined in terms of their broader clinical applications^6^, ^7^, ^8^, ^9^, ^10^, ^11^; however, follitropin δ is emerging as a promising alternative.

Follitropin δ offers a unique approach by standardizing the gonadotropin dosage based on body weight and ovarian reserve.^7^ This standardized dosing sets it apart from traditional practices that adjust dosages according to ovarian responsiveness, offering advantages in terms of reproducibility and ease of application.^7^ However, despite increasing research into follitropin δ,^12^, ^13^, ^14^, ^15^, ^16^, ^17^, ^18^, ^19^, ^20^, ^21^, ^22^, ^23^ a comprehensive comparison with the existing agents, follitropin α and β, is still lacking.

The current meta‐analysis aimed to evaluate the efficacy and safety of follitropin δ, especially with respect to its impacts on live birth rates and the incidence of moderate or severe OHSS. By examining the efficacy and safety of follitropin δ from multiple perspectives, this study aimed to support refinements in clinical practice and improve patient care in reproductive medicine.

## 2 METHODS

### 2.1 Protocol and registration

The protocol was registered on the Open Science Framework (OSF.IO; https://osf.io/2ghrx) on December 13, 2022. We adhered to the Preferred Reporting Items for Systematic Reviews and Meta‐Analysis 2020 (PRISMA‐2020) statement (Data S1).^24^

### 2.2 Eligibility criteria and participants

We searched for studies of randomized controlled trials (RCTs) that evaluated follitropin α/β and follitropin δ in controlled ovarian stimulation (COS). We included all types of articles, including peer‐reviewed papers, conference abstracts, and letters. We did not exclude studies based on language, geographical origin, duration of observation, or year of publication. We included RCTs with eligible participants consisting of women over 18 years old, of any ethnicity, who were undergoing COS with recombinant follicle‐stimulating hormone (r‐FSH), urinary‐FSH (u‐FSH), urinary human menopausal gonadotropin (u‐HMG), or follitropin δ.

### 2.3 Interventions and comparators

COS cycles using follitropin δ as part of a GnRH antagonist protocol or long GnRH agonist protocol were considered as interventions, and COS cycles using conventional r‐FSH, u‐FSH, or u‐HMG as part of a GnRH antagonist protocol or a long GnRH agonist protocol were considered as controls.

### 2.4 Outcomes of interest

The primary outcomes were live birth rates and the incidence of moderate or severe OHSS, classified by Golan's criteria.^25^ The secondary outcomes comprised the number of oocytes retrieved, ongoing pregnancy rates, number of blastocysts, and adverse drug reactions.

### 2.5 Information sources and study selection

We searched for publications in MEDLINE (by PubMed) since 1946, Embase (by ProQuest) since 1974, and the CENTRAL, ClinicalTrials.gov, and World Health Organization–International Clinical Trials Registry Platform (WHO‐ICTRP) databases from inception to December 14, 2022 (Table S1), using specific keywords. We contacted the first authors for unreported or supplemental data. Two reviewers (SK and TT) separately screened the titles and abstracts of all articles found in the search. Articles selected for abstract screening underwent a full‐text evaluation to determine their appropriateness. Any disagreements were resolved through conversation and consultation with a third reviewer (JW).

### 2.6 Data collection

Two independent reviewers (SK and TT) collected the following information from the studies: the authors' names, year of publication, study design, duration, follow‐up period, registry number, country, setting, inclusion/exclusion criteria, sample size, interventions, outcomes, and funding source.

### 2.7 Risk of bias and certainty of evidence assessment

In our summary of findings (SoF), we critically evaluated both the risk of bias and the certainty of the evidence. Two independent reviewers (SK and TT) evaluated the risk of bias using the Cochrane Risk of Bias 2 tool.^26^ The certainty of the evidence was assessed by two independent reviewers (SK and KK) using the grading of recommendations assessment, development, and evaluation (GRADE) approach.^27^ Any discrepancies between the reviewers were resolved through discussion. If a consensus could not be reached, a third reviewer (JW) served as an adjudicator.

### 2.8 Summary measures and synthesis of results

Primary summary measures were risk ratios (RRs) for binary outcomes and mean differences (MDs) for continuous outcomes. RRs were calculated and pooled with 95% confidence intervals (CIs) for the following binary variables: live birth, incidence of moderate or severe OHSS, incidence of moderate or severe early OHSS, ongoing pregnancy, and incidence of adverse drug reaction. MDs, along with their 95% CIs, were calculated and pooled for continuous outcomes including the number of retrieved oocytes and the number of blastocysts.

We performed the meta‐analysis using random‐effects models with Review Manager software (RevMan 5.4.1; the Cochrane Collaboration, Copenhagen, Denmark). No imputation of missing values was performed for continuous data, as recommended by the Cochrane Handbook.^25^ If missing data were suspected in an article, we contacted the authors for clarification.

Statistical heterogeneity was evaluated by visual inspection of the point estimates of overall RRs or MDs with 95% CIs, and by *I* ^2^ statistics. *I* ^2^ values were interpreted as follows: 0%–40% (possibly not important), 30%–60% (possible moderate heterogeneity), 50%–90% (possible substantial heterogeneity), and 75%–100% (considerable heterogeneity).^28^

### 2.9 Additional analyses

The original protocol outlined plans for handling ‘unit of analysis issues’, specifically implementation of cluster randomization at the unit level, randomized cross‐over studies, and multiple comparisons. However, these approaches were not executed because there were no cluster RCTs or cross‐over studies. We initially planned to perform subgroup analyses based on age (cut‐off 35 years) and anti‐Müllerian hormone (AMH) levels (cut‐off, 15 pmol/mL)^12^, ^13^, ^17^, ^18^, ^22^, ^23^, ^29^; however, no age‐specific analyses were available, and analyses based on AMH levels were confined to the number of retrieved oocytes. Therefore, a comprehensive subgroup analysis was not feasible. Similarly, we intended to perform sensitivity analyses, excluding studies that used imputed statistics and those that dealt with data from both the first and second halves of cross‐over RCTs. However, no studies met these criteria; therefore, the analyses were precluded.

### 2.10 Differences between study protocol and review

Our initial protocol identified the ‘incidence of moderate or severe OHSS’ as a primary outcome; however, further analysis indicated that OHSS could be stratified into early OHSS, occurring within 9 days post‐final maturation, and late OHSS, which manifested later.^30^ While late OHSS can often be mitigated by canceling embryo transfers based on presenting abdominal symptoms,^31^, ^32^ early OHSS is intrinsically linked to the controlled ovarian stimulation process.^33^, ^34^, ^35^, ^36^, ^37^ Therefore, we decided to include early OHSS as an additional outcome for evaluation in this study.

Regarding the practical clinical application of follitropin δ, we have experienced extremely low oocyte retrieval, particularly in cases with high ovarian reserve, raising concerns about diminished patient satisfaction. We aimed to address this issue by performing additional analyses of retrieved oocyte counts in patients with high and low ovarian reserves, respectively. The included studies established a cut‐off for AMH levels of 15 pmol/mL^12^, ^13^, ^17^, ^18^, ^22^, ^23^; therefore, we adopted this value in our review.

## 3 RESULTS

### 3.1 Study selection

**FIGURE 1:**
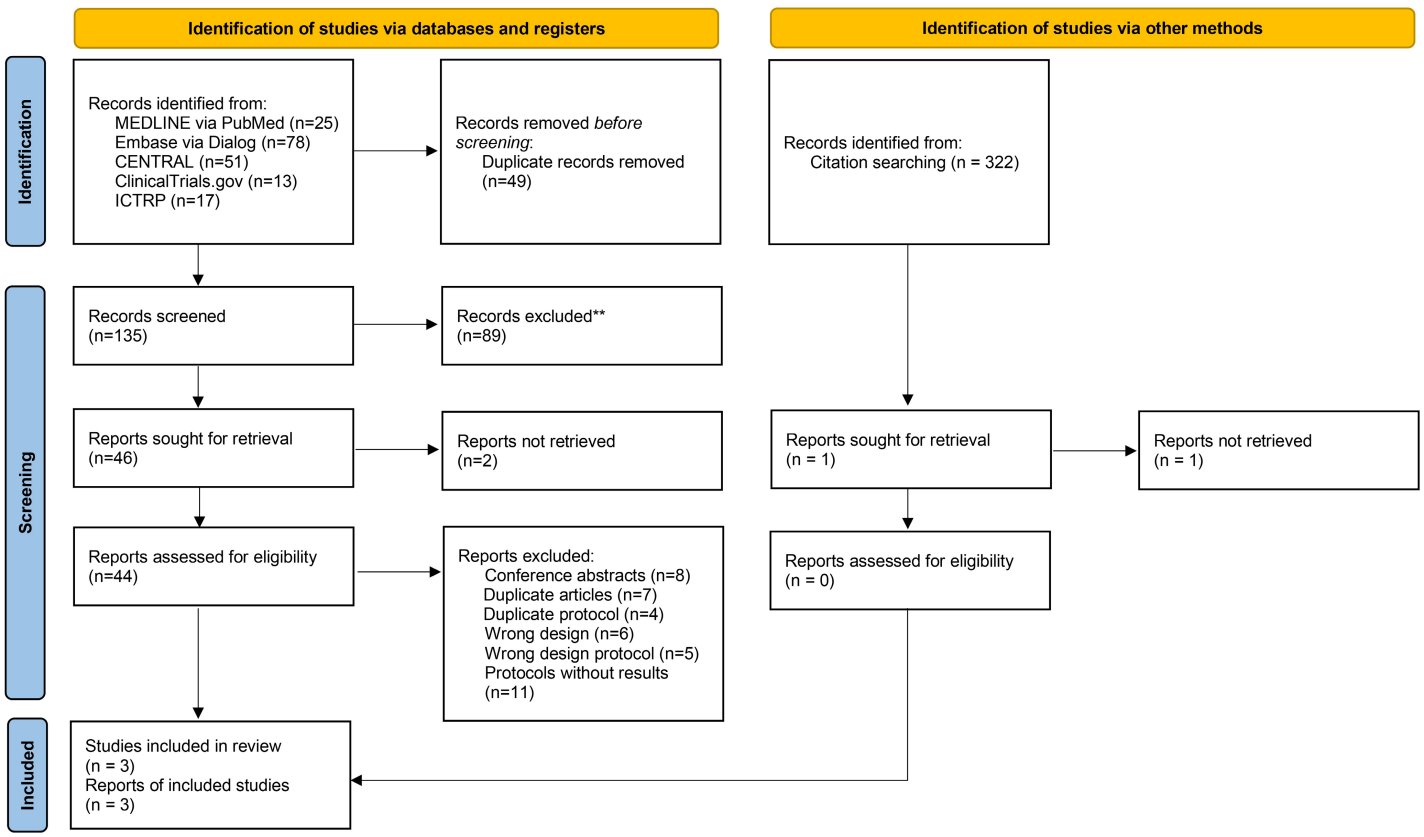
Preferred Reporting Items for Systematic Reviews and Meta‐Analysis (PRISMA) 2020 flow diagram. From: Page et al.^24^ For more information, visit: http://www.prisma‐statement.org/. CENTRAL, Cochrane Central Register of Controlled Trials; ICTRP, International Clinical Trials Registry Platform.

Our initial search in December 2022 identified 184 records. After removing duplicates, 135 records were screened for eligibility; 89 records were excluded through the title and abstract screening process, and 2 reports could not be obtained. Forty‐four articles underwent full‐text review, and three studies met the inclusion criteria and were included in the final review. Eleven protocols had no posted results (NCT05263388, EUCTR2021‐001785‐38, NCT04773353, NCT03738618, NCT03740737, NCT03809429, CTRI/2021/04/032835, EUCTR2017‐002783‐40‐AT, EUCTR2021‐001785‐38‐ES, NCT05403476, and NCT05263388). The entire study selection process is illustrated in the PRISMA flow diagram (Figure 1).

### 3.2 Study characteristics

**FIGURE 2:**
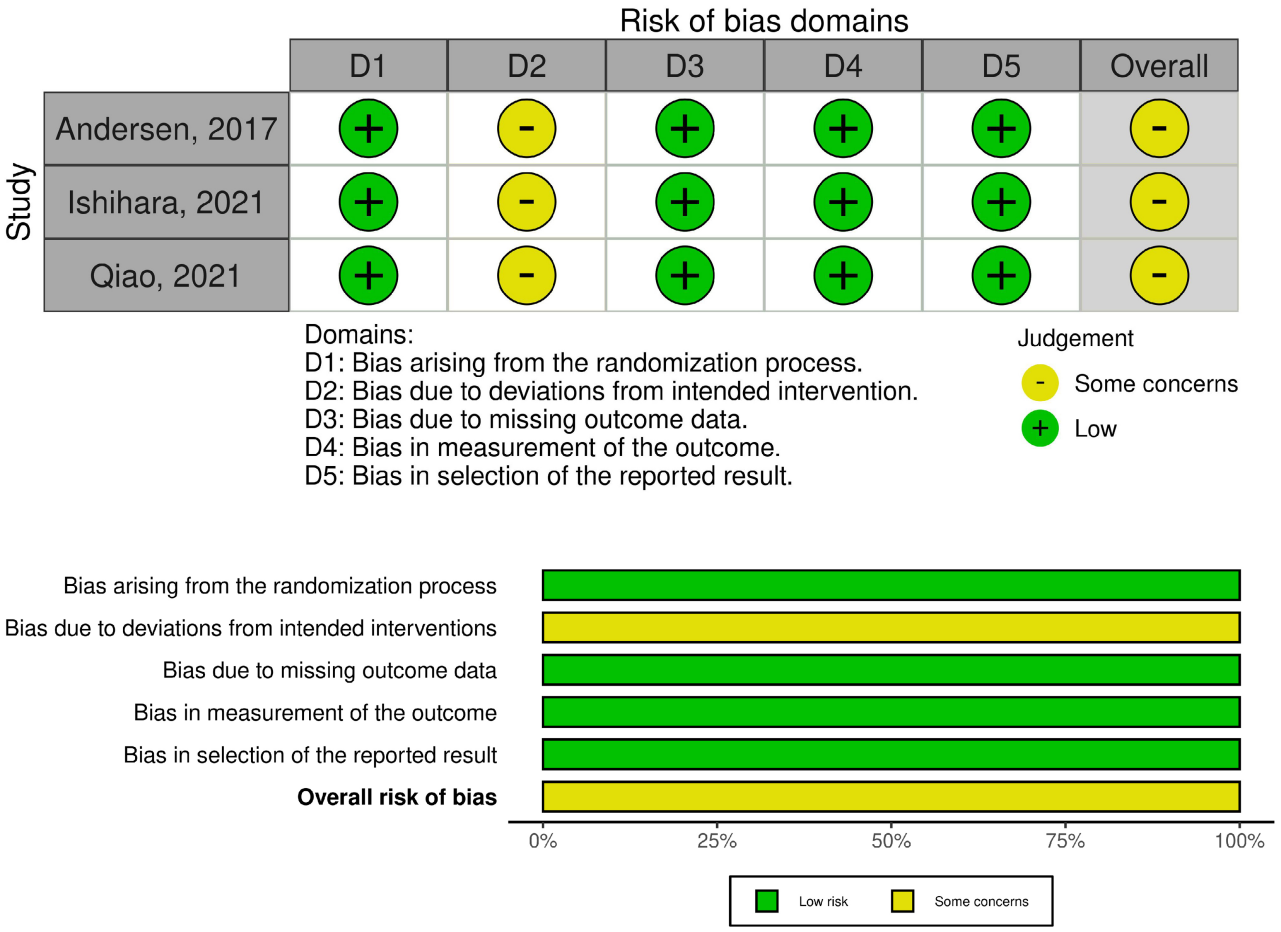
Traffic light and summary plots of risk of bias in live birth rates across the included studies.

The three included RCTs comprised 2682 participants and were performed in multiple international centers. The characteristics of the included studies are summarized in Table 1 and Table S2. The potential risk of bias for live birth rates in the quantitative synthesis is illustrated in Figure 2. The risk of bias for each outcome other than live birth rate is presented in Figure S1. All RCTs included in this study were funded by Ferring Pharmaceuticals, which manufactures follitropin δ (Rekovelle pen for subcutaneous injection 12/36/72 μg; Ferring Pharmaceuticals, Saint‐Prex, Switzerland).

**TABLE 1:** Characteristics of included studies.

Author year	Methods				Participants								Interventions		Outcomes		Note
	Study design	Duration of study	Follow‐up period	Registry number	Country	Setting	Inclusion criteria	Exclusion criteria	Number	Mean age ± SD (year)	Median AMH (IQR) (pmol/L)	Mean BMI ± SD (kg/m^2^)	Treatment arm	Control arm	Primary	Secondary	Funding source
Andersen, et al. (2017)	RCT parallel	Oct 8, 2013, to May 11, 2015	To Jan 11, 2016	NCT01956110	International	Multicenter	Infertile women treated with IVF/ICSI. (see Table S2a for details)	Patients with backgrounds that may worsen IVF/ICSI pregnancy outcome (see Table S2a for details).	Overall (1326): Intervention (665); Control (661)	33.4 ± 3.9; 33.2 ± 3.9	16.3 (9.0–24.8); 16.0 (9.1–25.5)	23.7 ± 3.4; 23.3 ± 3.3	Follitropin delta	Follitropin alfa	Ongoing Pregnancy Rate Ongoing Implantation Rate	Live Birth Rate Proportion of OHSS Number of oocytes retrieved (see Table S2a for details)	Ferring Pharmaceuticals
Ishihara, et al. (2021)	RCT parallel	July 7, 2017, to Sep 11, 2018	Not reported	NCT03228680	Japan	Multicenter	Infertile women treated with IVF/ICSI. (see Table S2b for details)	Patients with backgrounds that may worsen IVF/ICSI pregnancy outcome (see Table S2b for details).	Overall (347): Intervention (170); Control (177)	34.2 ± 3.5; 34.0 ± 3.4	18.2 (11.0–28.2); 16.7 (11.3–27.4)	21.4 ± 2.7; 21.6 ± 2.8	Follitropin delta	Follitropin beta	Number of oocytes retrieved	Ongoing Pregnancy Rate Ongoing Implantation Rate Live Birth Rate Proportion of OHSS (see Table S2b for details)	Ferring Pharmaceuticals
Qiao, et al. (2021)	RCT parallel	Dec 1, 2017, to Jan 3, 2020	To Sep 1, 2020	NCT03296527	International	Multicenter	Infertile women treated with IVF/ICSI (see Table S2c for details).	Patients with backgrounds that may worsen IVF/ICSI pregnancy outcome (see Table S2c for details).	Overall (1009): Intervention (499); Control (510)	31.1 ± 3.7; 31.2 ± 3.8	23.4 (16.1–32.9); 22.6 (15.3–33.2)	21.8 ± 2.7; 21.8 ± 2.8	Follitropin delta	Follitropin alfa	Ongoing Pregnancy Rate	Ongoing Implantation Rate Live Birth Rate Proportion of OHSS Number of oocytes retrieved (see Table S2c for details)	Ferring Pharmaceuticals

Abbreviations: AMH, anti‐Müllerian hormone; BMI, body mass index; ICSI, intra‐cytoplasmic sperm injection; IQR, interquartile range; IVF, in vitro fertilization; OHSS, ovarian hyperstimulation syndrome; RCT, randomized controlled trial; SD, standard deviation.

### 3.3 SoF

We summarized the certainty of evidence based on the GRADE approach and the results of the key outcomes in the SoF table (Table 2). The certainties of evidence for the live birth rate and the incidence of moderate or severe OHSS were both rated as low. A detailed interpretation and assessment of the certainty of evidence is presented in Table 2.

**TABLE 2:** Summary of findings.

Follitropin δ compared with Follitropin α/β for ART treatment						
Patient or population: Women aged under 40 undergoing their first IVF/ICSI cycle.						
Setting: Outpatients						
Intervention: Follitropin δ						
Comparison: Follitropin α/β						
Outcomes	Anticipated absolute effects^a^ (95% CI)		Relative effect (95% CI)	No of participants (studies)	Certainty of the evidence (GRADE)	Comments
	Risk with Follitropin α/β	Risk with Follitropin δ				
Live birth rate	269 per 1000	302 per 1000 (245–372)	RR 1.12 (0.91–1.38)	2682 (3 RCTs)	⨁⨁◯◯ Low^b^ ^,^ ^c^	Follitropin δ may result in little to no difference in the live birth rate.
Incidence of moderate or severe OHSS	57 per 1000	45 per 1000 (27–72)	RR 0.78 (0.48–1.26)	2682 (3 RCTs)	⨁⨁◯◯ Low^b^ ^,^ ^c^	Follitropin δ may result in little to no difference in the incidence of moderate or severe OHSS.
Incidence of moderate or severe early OHSS	42 per 1000	29 per 1000 (19–43)	RR 0.69 (0.46–1.04)	2682 (3 RCTs)	⨁⨁⨁◯ Moderate^c^	Follitropin δ likely reduces incidence of moderate or severe early OHSS slightly.
Number of retrieved oocytes		MD 1.17 lower (1.64 lower to 0.7 lower)	—	2682 (3 RCTs)	⨁⨁⨁◯ Moderate^c^	Follitropin δ likely reduces number of retrieved oocytes.
Ongoing pregnancy rate	277 per 1000	305 per 1000 (255–361)	RR 1.10 (0.92–1.30)	2682 (3 RCTs)	⨁⨁◯◯ Low^b^ ^,^ ^c^	Follitropin δ may result in little to no difference in ongoing pregnancy rate.
Number of blastocysts		MD 0.61 lower (1.48 lower to 0.27 higher)	—	1673 (2 RCTs)	⨁⨁◯◯ Low^c^ ^,^ ^d^	Follitropin δ may reduce number of blastocysts slightly.
Incidence of adverse drug reactions	256 per 1000	248 per 1000 (212–292)	RR 0.97 (0.83–1.14)	2682 (3 RCTs)	⨁⨁◯◯ Low^b^ ^,^ ^c^	Follitropin δ may result in little to no difference in incidence of adverse drug reactions.

Abbreviations: ART, assisted reproductive technology; CI, confidence interval; ICSI, intra‐cytoplasmic sperm injection; IVF, in vitro fertilization; MD, mean difference; OHSS, ovarian hyperstimulation syndrome; RCT, randomized controlled trial; RR, risk ratio.

GRADE Working Group grades of evidence

High certainty: We are very confident that the true effect lies close to that of the estimate of the effect.

Moderate certainty: We are moderately confident in the effect estimate: the true effect is likely to be close to the estimate of the effect, but there is a possibility that it is substantially different.

Low certainty: Our confidence in the effect estimate is limited: the true effect may be substantially different from the estimate of the effect.

Very low certainty: We have very little confidence in the effect estimate: the true effect is likely to be substantially different from the estimate of effect.

^a^ The risk in the intervention group (and its 95% confidence interval) is based on the assumed risk in the comparison group and the relative effect of the intervention (and its 95% CI).

^b^ Based on variable results among reports, the grade was reduced by one level due to inconsistency.

^c^ Based on the 95% confidence interval of risk difference, the grade was reduced by one level due to imprecision.

^d^ Based on only two included RCTs, the grade was reduced by one level due to imprecision.

### 3.4 Primary outcomes

#### 3.4.1 Live birth rate

**FIGURE 3:**
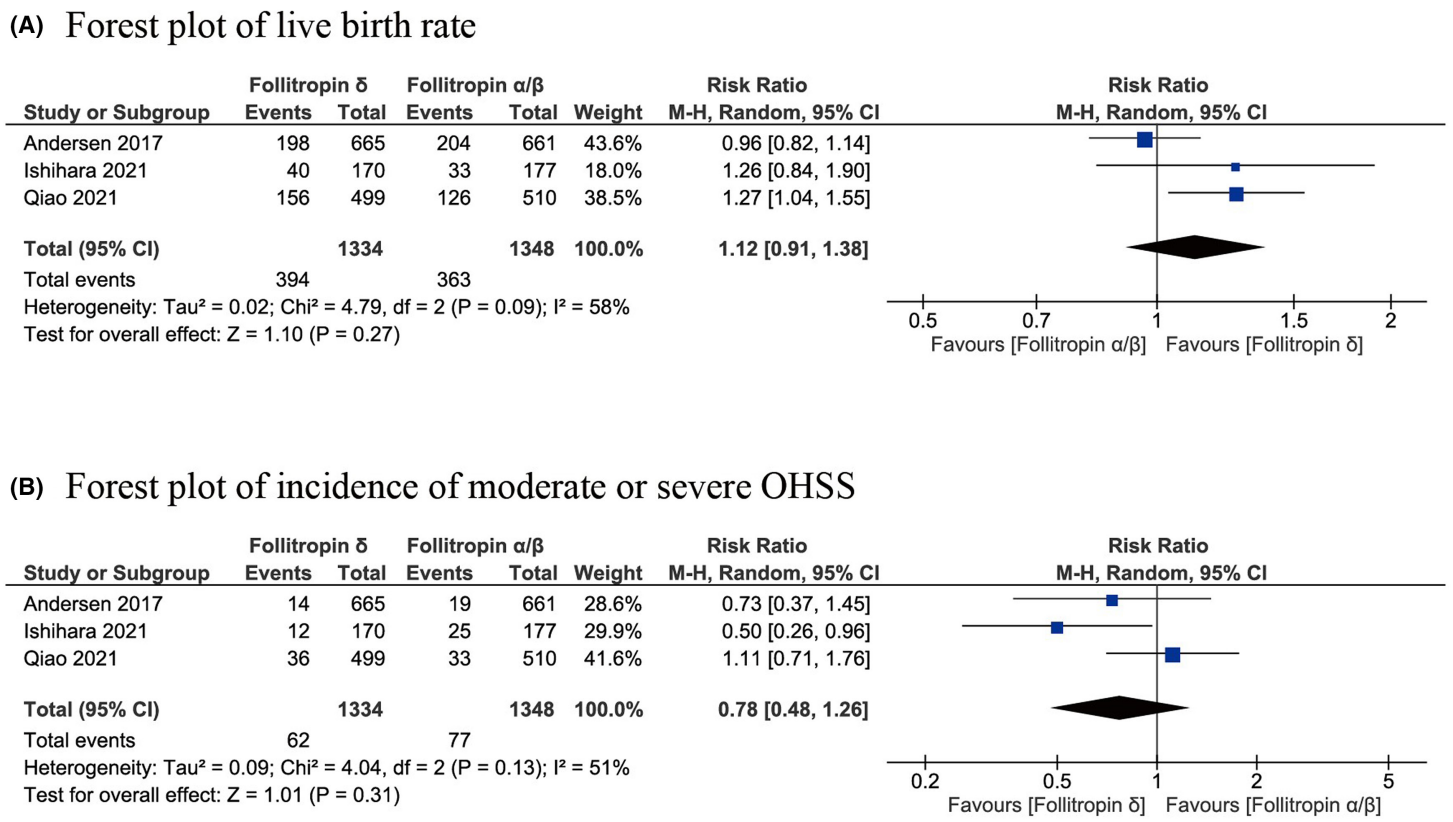
Forest plots of the primary outcomes. Forest plots of (A) live birth rate and (B) incidence of moderate or severe OHSS. CI, confidence interval; df, degrees of freedom; M–H, Mantel–Haenszel.

Our meta‐analysis integrated data from three RCTs, involving 2682 participants, to assess the impact of follitropin δ on live birth rates. The synthesized data suggested that follitropin δ may result in little to no difference in the live birth rate compared with follitropin α/β (RR, 1.12; 95% CI, 0.91–1.38; *I* ^2^ = 58%; certainty of evidence, low) (Figure 3A).

#### 3.4.2 Incidence of moderate or severe OHSS

Our meta‐analysis integrated data from two RCTs, involving 2682 participants, to assess the impact of follitropin δ on the incidence of moderate or severe OHSS. The synthesized data indicated that follitropin δ may result in little to no difference in the incidence of moderate or severe OHSS compared with follitropin α/β (RR, 0.78; 95% CI, 0.48–1.26; *I* ^2^ = 51%; certainty of evidence, low) (Figure 3B).

### 3.5 Secondary outcomes

#### 3.5.1 Incidence of moderate or severe early OHSS

**FIGURE 4:**
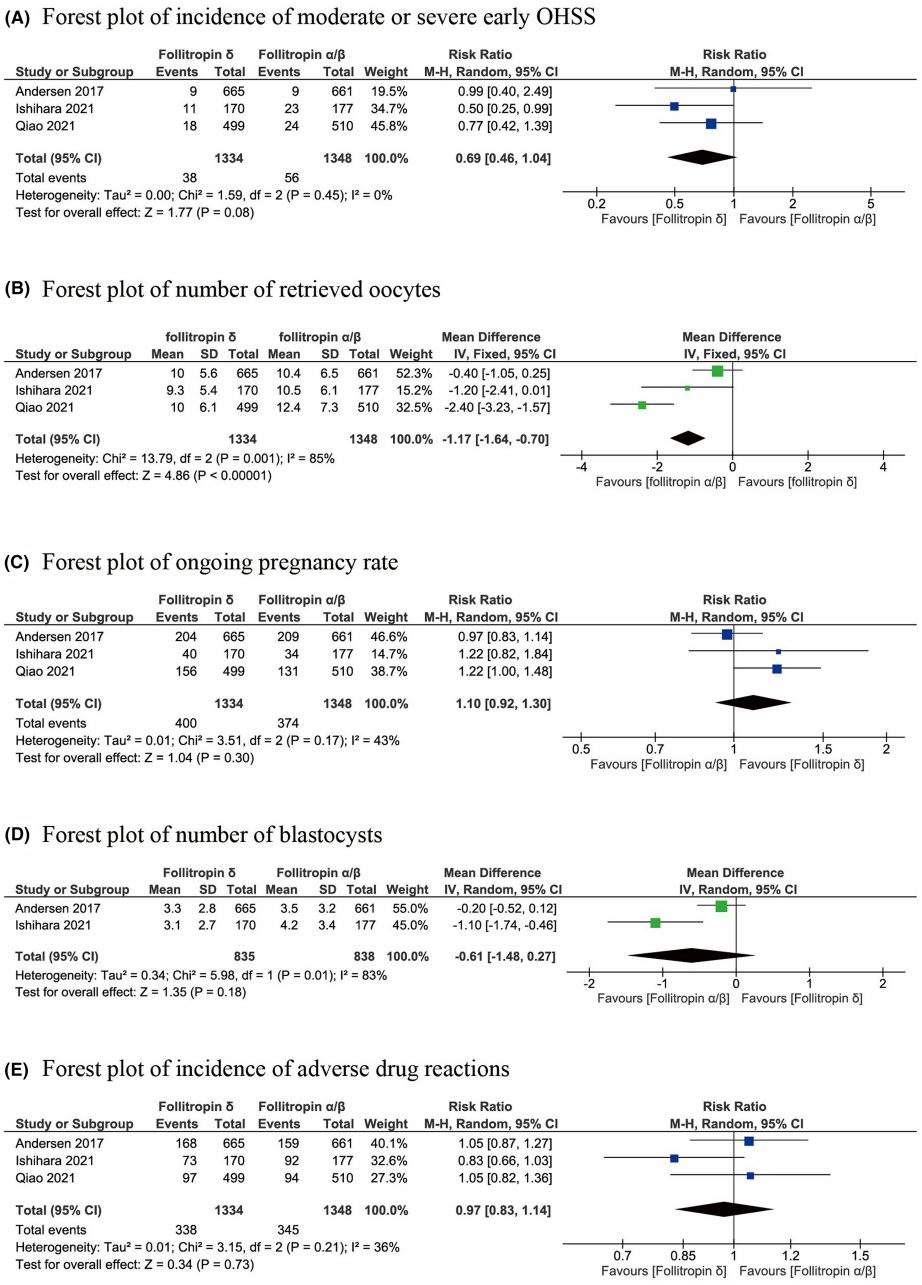
Forest plots of secondary outcomes. Forest plots of (A) incidence of moderate or severe early OHSS, (B) number of retrieved oocytes, (C) ongoing pregnancy rate, (D) number of blastocysts, and (E) incidence of adverse drug reactions. CI, confidence interval; df, degrees of freedom; IV, inverse variance; M–H, Mantel–Haenszel; SD, standard deviation.

Our meta‐analysis integrated data from three RCTs, involving 2682 participants, to assess the impact of follitropin δ on the incidence of moderate or severe early OHSS. The synthesized data indicated that follitropin δ slightly reduced the incidence of moderate or severe early OHSS compared with follitropin α/β (RR, 0.69; 95% CI, 0.46–1.04; *I* ^2^ = 0%; certainty of evidence, moderate) (Figure 4A).

#### 3.5.2 Number of retrieved oocytes

Our meta‐analysis integrated data from three RCTs, involving a total of 2682 participants, to assess the impact of follitropin δ on the number of retrieved oocytes. The synthesized data indicated that follitropin δ reduced the number of retrieved oocytes compared with follitropin α/β (MD, −1.17; 95% CI, −1.64 to −0.70; *I* ^2^ = 85%; certainty of evidence, moderate) (Figure 4B).

#### 3.5.3 Ongoing pregnancy rate

Our meta‐analysis integrated data from three RCTs, involving 2682 participants, to assess the impact of follitropin δ on ongoing pregnancy rates. The synthesized data suggested that follitropin δ had little or no effect on ongoing pregnancy rates compared with follitropin α/β (RR, 1.10; 95% CI, 0.92–1.30; *I* ^2^ = 43%; certainty of evidence, low) (Figure 4C).

#### 3.5.4 Number of blastocysts

One study was excluded from the meta‐analysis due to its focus on the rates of split‐stage embryo attainment, without providing data on blastocyst attainment. Thus, our meta‐analysis integrated data from two RCTs, involving 1673 participants, to assess the impact of follitropin δ on the number of blastocysts. The synthesized data indicated that follitropin δ had little or no effect on blastocyst numbers compared with follitropin α/β (MD, −0.61; 95% CI, −1.48 to 0.27; *I* ^2^ = 83%; certainty of evidence, low) (Figure 4D).

#### 3.5.5 Incidence of adverse drug reactions

One of the studies included in the review did not report the incidence of adverse drug reactions. However, the outcomes were publicly available in the registered protocol; therefore, we cited the findings from the protocol. Our meta‐analysis integrated data from three RCTs, involving 2682 participants, to assess the impact of follitropin δ on the incidence of adverse drug reactions. The synthesized data suggested that follitropin δ had little or no effect on the incidence of adverse drug reactions compared with follitropin α /β (RR, 0.97; 95% CI, 0.83–1.14; *I* ^2^ = 36%; certainty of evidence, low) (Figure 4E).

### 3.6 Additional analyses

For the outcome measure of retrieved oocytes, we performed a subgroup analysis to investigate the impact of follitropin δ usage on the number of retrieved oocytes in groups with different ovarian reserves, using an AMH cut‐off value of 15 pmol/mL.

**FIGURE 5:**
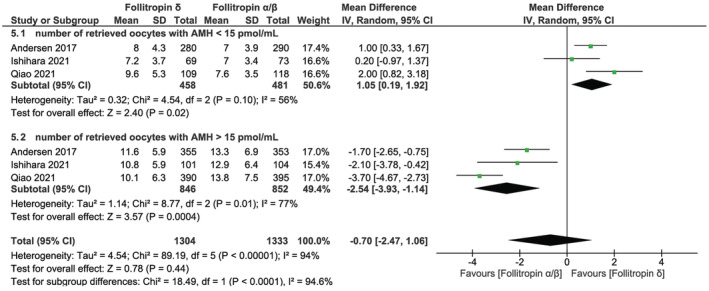
Forest plot of the number of retrieved oocytes stratified by an AMH cut‐off value of 15 pmol/mL. CI, confidence interval; df, degrees of freedom; IV, inverse variance; SD, standard deviation.

Our meta‐analysis integrated data from three RCTs to assess the impact of follitropin δ on the number of retrieved oocytes stratified by AMH levels, with a cut‐off value of 15 pmol/mL. Compared with follitropin α/β, follitropin δ resulted in a large increase in the number of retrieved oocytes in the subgroup with AMH levels <15 pmol/mL, involving 939 participants (MD, 1.05; 95% CI, 0.19–1.92; *I* ^2^ = 56%), but a large reduction in the number of retrieved oocytes in the subgroup with AMH levels ≥15 pmol/mL, comprising 1695 participants (MD, −2.54; 95% CI, −3.93 to −1.14; *I* ^2^ = 77%) (Figure 5).

## 4 DISCUSSION

Our systematic review and meta‐analysis collated data from three RCTs involving 2682 participants to evaluate the efficacy and safety of follitropin δ in COS as part of ART.^12^, ^13^, ^17^, ^18^, ^22^, ^23^ Notably, follitropin δ did not significantly differ in improving the live birth rates or in reducing the incidence of moderate or severe OHSS compared with existing follitropin α/β formulations. Therefore, while follitropin δ was expected to yield clinical outcomes similar to follitropin α/β, our data did not conclusively prove follitropin δ's clinical superiority in ART.

This study was the first systematic review and meta‐analysis to directly compare follitropin α/β with follitropin δ, analyzing focused datasets from RCTs to assess the efficacy and safety of follitropin δ. Traditional follitropin α/β protocols often require clinician‐led adjustments based on factors such as follicular growth and abdominal symptoms,^38^, ^39^ and this variability not only introduces clinical inconsistency but also increases the risk of OHSS.^40^, ^41^ In contrast, follitropin δ is administered using a more standardized approach based on objective criteria including body weight and ovarian reserve; thus potentially minimizing clinician‐introduced variability.^6^ Our analysis suggests that follitropin δ may achieve more consistent treatment outcomes through a standardized dosing algorithm while maintaining comparable live birth rates. These results become increasingly relevant as ART gains wider acceptance, suggesting that comparable results could be achieved without relying heavily on individual clinician expertise.

Our systematic review and meta‐analysis demonstrated that follitropin δ may offer a safer approach to COS by reducing the incidence of early‐onset OHSS without compromising live birth rates. Typically, to prevent early‐onset OHSS before final maturation in COS, clinicians opt for antagonist protocols over agonist protocols.^42^, ^43^ Moreover, post‐final maturation early‐onset OHSS prevention, options include using GnRH agonist nasal sprays for final maturation or administering medications like cabergoline, metformin, and plasma volume expansion^4^, ^44^, ^45^, ^46^ following oocyte retrieval. Follitropin δ could be a novel option to prevent early‐onset OHSS prior to the final maturation in COS, specifically when using an antagonist protocol. In addition to its clinical utility, follitropin δ is associated with a reduced number of retrieved oocytes, probably as a result of its algorithmic dosing strategy. Prior studies established an optimal oocyte retrieval range of 5–15 for the best outcomes in fresh embryo transfer cycles.^47^ The ability of follitropin δ to maintain oocyte counts within this clinically beneficial range could thus enhance the safety of COS cycles, particularly given the increasing risk of severe OHSS when ≥18 oocytes are retrieved.^48^ Thus, follitropin δ not only represents a promising strategy for reducing OHSS but also aligns with a targeted and safer oocyte retrieval strategy. These factors suggest that follitropin δ may be a clinically viable option for inclusion in treatment algorithms within infertility management.

Nevertheless, the possibility that COS cycles using follitropin δ may result in fewer retrieved oocytes and acquired blastocysts compared with cycles using follitropin α/β remains a clinical concern. There have been insurance limitations on embryo transfer in Japan since April 2022, with emphasis on improving pregnancy rates per treatment cycle,^49^ and one strategy for enhancing pregnancy rates is to increase the number of embryos transferred.^50^, ^51^, ^52^, ^53^ Moreover, a multicenter study involving approximately 15 000 individuals showed that the cumulative live birth rate, including frozen–thawed embryo transfer cycles, reliably increased with increasing number of oocytes retrieved, reaching up to 70% when ≥25 oocytes were obtained.^54^, ^55^ However, apart from these considerations, it is crucial to maintain low rates of twin and multiple pregnancies to improve perinatal outcomes and reduce the burden on perinatal medical care.^56^, ^57^ Therefore, single embryo transfer is generally recommended, in line with Japanese guidelines, with a limit of two embryos under specific conditions, such as repeated failure or in women aged >35 years.^58^, ^59^, ^60^ Improving pregnancy rates while adhering to the single embryo transfer paradigm requires precise embryo selection via non‐invasive methods, such as in vitro blastocyst culture,^61^, ^62^ or invasive techniques like pre‐implantation genetic testing for aneuploidy.^63^, ^64^

The current subgroup analysis suggested that the use of follitropin δ may present challenges in high responders. Specifically, follitropin δ could be beneficial in patients with a low ovarian reserve (defined by a cut‐off of AMH = 15 pmol/mL, equivalent to 2.1 ng/mL), potentially increasing the number of retrieved oocytes; however, with a decrease in retrieved oocytes in women with a high ovarian reserve. There are two possible reasons for this caution. First, follitropin α/β is synthesized from Chinese hamster ovary cell lines, whereas follitropin δ is derived from human cell lines, leading to different metabolic pathways in vivo that could influence the ovarian response.^6^ Second, the dosing algorithm for follitropin δ may be configured to administer a smaller dosage for high responders, thereby affecting the overall number of oocytes retrieved and consequently limiting the pool of embryos available for precise selection methods. These differences are not directly attributable to the pharmacological effects of follitropin δ and follitropin α/β, as they are influenced by the respective dosing algorithms. However, these observations represent the actual responses observed with each formulation using the approved dosing regimen.

In terms of safety, our meta‐analysis suggested that follitropin δ had little or no effect on the incidence of adverse drug reactions compared with follitropin α/β formulations. Specifically, minor adverse events, such as pain at the injection site, lower abdominal discomfort, and pelvic pain, occurred at comparable rates in the two treatment groups. Although self‐injection is an integral component of COS, it can be an invasive and stressful process,^65^ particularly in patients with a needle phobia, potentially compromising treatment adherence.^66^ Notably, as of September 2023, follitropin δ has only been available in Japan as pen‐type formulations, while follitropin α is available as both vial and pen‐type options and follitropin β is distributed solely in vial form. The less‐invasive pen‐type formulations of follitropin δ^67^ align well with patient needs, offering a potential advantage in terms of reducing patient burden.

Although this study used data from multicenter international trials, its generalizability is restricted by several key limitations. First, we focused solely on live birth rates following fresh embryo transfers, without considering the cumulative number of children per oocyte retrieval. This is particularly relevant given that family planning does not aim for just a single birth. Second, the applicability of our findings to the Japanese ART landscape is questionable. According to the latest statistics in Japan, 87.8% of all embryo transfer cycles involved the use of frozen–thawed embryos^68^; therefore, the effectiveness of follitropin δ in the predominant ART practices in Japan remains uncertain. Further studies are needed to broaden the scope to include frozen–thawed embryo transfers and to examine the impact of follitropin δ on cumulative birth outcomes to establish more comprehensive clinical guidelines. Third, our study population was limited to women under 40 years, which is inconsistent with the real‐world demographics in Japan, where the most common age range for ART treatment is 39–43 years.^68^ Additionally, the background data on follitropin δ dosage, such as weight and body mass index, do not align with the Japanese demographic profile, which tends to exhibit leaner body types compared with Western populations (Table 1). These differences in age and demographic characteristics might impair the external validity of our findings. Finally, it is important to note that all three of the included RCTs were supported financially by the drug manufacturer, Ferring Pharmaceuticals. This not only raises concerns about affiliation bias due to the uniform funding source but also highlights the limited number of studies in our analysis. Although industry funding does not inherently introduce publication bias,^69^ the implications of such funding on our study's conclusions should be carefully considered.

This systematic review and meta‐analysis showed that follitropin δ may result in little to no difference in the live birth rates or the incidence of moderate or severe OHSS compared with existing follitropin α/β formulations. These findings suggest that follitropin δ could serve as an alternative to follitropin α/β in COS cycles in ART, offering comparable therapeutic benefits. This is particularly promising owing to the potentially safe administration, regardless of physician experience. However, notably, follitropin δ may not be advantageous in women with high ovarian reserve. While follitropin δ could offer safety benefits in preventing OHSS in high ovarian reserve cases, there may also be drawbacks, such as decreased oocyte retrieval and blastocyst formation rates when following the prescribed protocols for follitropin δ. Therefore, it is essential to assess each case carefully to determine the appropriate medication.

## CONFLICT OF INTEREST STATEMENT

The authors declare no conflict of interest for this article.

## Supporting information


Figure S1.

Table S1.

Table S2.

Data S1.
