# Supplementary material for: Efficacy and safety of follitropin delta versus follitropin alpha/beta in infertility treatment: A systematic review and meta‐analysis
Source: Reprod Med Biol. 2024 Mar 25;23(1):e12573. doi: 10.1002/rmb2.12573 (PMC10961712; doi:10.1002/rmb2.12573)
Supplement: Supplementary file 2 — Table S1. [file RMB2-23-e12573-s004.docx]

**Supplementary table S1. Detail of search formulas**

1. Search formula for MEDLINE via PubMed

| #1 | "Fertilization in Vitro"[mh] |
| --- | --- |
| #2 | "Oocyte Retrieval"[mh] |
| #3 | "Infertility"[mh] |
| #4 | "in vitro fertilization"[tiab] |
| #5 | ivf[tiab] |
| #6 | "intracytoplasmic sperm injection"[tiab] |
| #7 | icsi[tiab] |
| #8 | "oocyte pick up"[tiab] |
| #9 | "oocyte retrieval"[tiab] |
| #10 | "egg retrieval"[tiab] |
| #11 | "ovum pick up"[tiab] |
| #12 | "ovum retrieval"[tiab] |
| #13 | #1 OR #2 OR #3 OR #4 OR #5 OR #6 OR #7 OR #8 OR #9 OR #10 OR #11 OR #12 |
| #14 | "follitropin delta"[Title/Abstract:~5] |
| #15 | "REKOVELLE"[tiab] |
| #16 | #14 OR #15 |
| #17 | #13 AND #16 |

1. Search formula for CENTRAL

| #1 | [mh "Fertilization in Vitro"] |
| --- | --- |
| #2 | [mh "Oocyte Retrieval"] |
| #3 | [mh Infertility] |
| #4 | "in vitro fertilization":ti,ab |
| #5 | ivf:ti,ab |
| #6 | "intracytoplasmic sperm injection":ti,ab |
| #7 | icsi:ti,ab |
| #8 | "oocyte pick up":ti,ab |
| #9 | "oocyte retrieval":ti,ab |
| #10 | "egg retrieval":ti,ab |
| #11 | "ovum pick up":ti,ab |
| #12 | "ovum retrieval":ti,ab |
| #13 | [mh "Fertilization in Vitro"] OR [mh "Oocyte Retrieval"] OR [mh Infertility] OR "in vitro fertilization":ti,ab OR ivf:ti,ab OR "intracytoplasmic sperm injection":ti,ab OR icsi:ti,ab OR "oocyte pick up":ti,ab OR "Oocyte Retrieval":ti,ab OR "egg retrieval":ti,ab OR "ovum pick up":ti,ab OR "ovum retrieval":ti,ab |
| #14 | "follitropin delta":ti,ab |
| #15 | REKOVELLE:ti,ab |
| #16 | "follitropin delta":ti,ab OR REKOVELLE:ti,ab |
| #17 | ([mh "Fertilization in Vitro"] OR [mh "Oocyte Retrieval"] OR [mh Infertility] OR "in vitro fertilization":ti,ab OR ivf:ti,ab OR "intracytoplasmic sperm injection":ti,ab OR icsi:ti,ab OR "oocyte pick up":ti,ab OR "Oocyte Retrieval":ti,ab OR "egg retrieval":ti,ab OR "ovum pick up":ti,ab OR "ovum retrieval":ti,ab) AND ("follitropin delta":ti,ab OR REKOVELLE:ti,ab) |

1. Search formula for Embase via ProQuest

| S1 | EMB.EXACT.EXPLODE("in vitro fertilization") |
| --- | --- |
| S2 | EMB.EXACT.EXPLODE("oocyte retrieval") |
| S3 | ab("Infertility") OR ti("Infertility") |
| S4 | ab("in vitro fertilization") OR ti("in vitro fertilization") |
| S5 | ab(ivf) OR ti(ivf) |
| S6 | ab("intracytoplasmic sperm injection") OR ti("intracytoplasmic sperm injection") |
| S7 | ab(icsi) OR ti(icsi) |
| S8 | ab("oocyte pick up") OR ti("oocyte pick up") |
| S9 | ab("oocyte retrieval") OR ti("oocyte retrieval") |
| S10 | ab("egg retrieval") OR ti("egg retrieval") |
| S11 | ab("ovum pick up") OR ti("ovum pick up") |
| S12 | ab("ovum retrieval") OR ti("ovum retrieval") |
| S13 | S1 OR S2 OR S3 OR S4 OR S5 OR S6 OR S7 OR S8 OR S9 OR S10 OR S11 OR S12 |
| S14 | ab("follitropin delta") OR ti("follitropin delta") |
| S15 | ab("REKOVELLE") OR ti("REKOVELLE") |
| S16 | S14 OR S15 |
| S17 | S13 AND S16 |

1. Search formula for ClinicalTrials.gov

| Condition or disease | infertility |
| --- | --- |
| Intervention | follitropin delta |
| Sex | female only |
| Age | over 18 |

1. Search formula for ICTRP

| #1 Conditions: | infertility |
| --- | --- |
| #2 Intervention: | follitropin delta |
| #3 | #1 AND #2 |
