# Supplementary material for: Efficacy and safety of follitropin delta versus follitropin alpha/beta in infertility treatment: A systematic review and meta‐analysis
Source: Reprod Med Biol. 2024 Mar 25;23(1):e12573. doi: 10.1002/rmb2.12573 (PMC10961712; doi:10.1002/rmb2.12573)
Supplement: Supplementary file 3 — Table S2. [file RMB2-23-e12573-s003.docx]

**Supplementary table S2(a)-(c). Details of inclusion/exclusion criteria and secondary outcomes in each study.**

1. Andersen, et al. (2017)

| Details of Inclusion criteria | 1. Informed consent documents signed before screening evaluations. 2. In good physical and mental health. 3. Premenopausal women between the ages of 18 and 40 years. The subjects must be at least 18 years (including the 18th birthday) when they sign the informed consent and no more than 40 years (up to the day before the 41st birthday) at the time of randomization. 4. Infertile women diagnosed with tubal infertility, unexplained infertility, endometriosis stage I/II or with partners diagnosed with male factor infertility, eligible for IVF and/or intracytoplasmic sperm injection (ICSI) using fresh or frozen ejaculated sperm from male partner or sperm donor. 5. Infertility for at least 1 year before randomization for subjects aged ≤37 years or for at least 6 months for subjects aged ≥38 years (not applicable in case of tubal or severe male factor infertility) 6. The trial cycle will be the subject's first controlled ovarian stimulation cycle for IVF/ICSI. 7. Regular menstrual cycles of 24–35 days (both inclusive), presumed to be ovulatory. 8. Hysterosalpingography, hysteroscopy, saline infusion sonography, or transvaginal ultrasound documenting a uterus consistent with expected normal function (e.g., no evidence of clinically interfering uterine fibroids defined as submucous or intramural fibroids larger than 3 cm in diameter, no polyps, and no congenital structural abnormalities that are associated with a reduced chance of pregnancy) within 1 year before randomization. 9. Transvaginal ultrasound documenting presence and adequate visualization of both ovaries, without evidence of significant abnormality (e.g., no endometrioma greater than 3 cm or enlarged ovaries that would contraindicate the use of gonadotropins) and normal adnexa (e.g., no hydrosalpinx) within 1 year before randomization. Both ovaries must be accessible for oocyte retrieval. 10. Early follicular phase (cycle day 2–4) serum levels of FSH between 1 and 15 IU/L (results obtained within 3 months before randomization). 11. Negative serum hepatitis B surface antigen, hepatitis C virus, and human immunodeficiency virus antibody tests within 2 years before randomization. 12. Body mass index between 17.5 and 32.0 kg/m2 (both inclusive) at screening. 13. If aged ≤37 years willing to accept single blastocyst transfer. If aged ≥38 years willing to accept transfer of a single good-quality blastocyst or double blastocyst transfer if no good-quality blastocyst is available. 14. Willing to accept transfer of maximum two blastocysts in cryopreserved cycles with blastocysts originating from the trial cycle and conducted within 1 year after randomization. |
| --- | --- |
| Details of Exclusion criteria | 1. Known endometriosis stage III–IV. 2. One or more follicles ≥10 mm observed on the transvaginal ultrasound before randomization on stimulation day. 3. Known history of recurrent miscarriage (defined as three consecutive losses after ultrasound confirmation of pregnancy (excluding ectopic pregnancy) and before week 24 of pregnancy). 4. Known abnormal karyotype of subject or of her partner/sperm donor, as applicable, depending on source of sperm used for insemination in this trial. In case partner sperm will be used and the sperm production is severely impaired (concentration <1 × 10^6^/mL), normal karyotype, including no Y-chromosome microdeletion, must be documented. 5. Any known clinically significant systemic disease (e.g., insulin-dependent diabetes). 6. Known inherited or acquired thrombophilia disease. 7. Active arterial or venous thromboembolism or severe thrombophlebitis, or a history of these events. 8. Known porphyria. 9. Any known endocrine or metabolic abnormalities (pituitary, adrenal, pancreas, liver or kidney) that can compromise participation in the trial with the exception of controlled thyroid function disease. 10. Known presence of anti-FSH antibodies (based on the information available in the subject's medical records; i.e., not based on the anti-FSH antibody analyses conducted in the trial). 11. Known tumors of the ovary, breast, uterus, adrenal gland, pituitary, or hypothalamus that would contraindicate the use of gonadotropins. 12. Known moderate or severe impairment of renal or hepatic function. 13. Currently breast-feeding. 14. Undiagnosed vaginal bleeding. 15. Known abnormal cervical cytology of clinical significance observed within 3 years before randomization (unless the clinical significance has been resolved). 16. Findings at the gynecologic examination at screening that preclude gonadotropin stimulation or are associated with a reduced chance of pregnancy (e.g., congenital uterine abnormalities or retained intrauterine device). 17. Pregnancy (negative urinary pregnancy tests must be documented at screening and before randomization) or contraindication to pregnancy. 18. Known current active pelvic inflammatory disease. 19. Use of fertility modifiers during the last menstrual cycle before randomization, including DHEA or cycle programming with oral contraceptives, progestogen, or estrogen preparations. 20. Use of hormonal preparations (except for thyroid medication) during the last menstrual cycle before randomization. 21. Known history of chemotherapy (except for gestational conditions) or radiotherapy. 22. Current or past (1 year before randomization) abuse of alcohol or drugs, and/or current (last month) intake of more than 14 units of alcohol per week. 23. Current or past (3 months before randomization) smoking habit of more than 10 cigarettes per day. 24. Hypersensitivity to any active ingredient or excipients in the medicinal products used in the trial. 25. Previous participation in the trial. 26. Use of any nonregistered investigational drugs during the last 3 months before randomization. |
| Details of secondary outcomes | 1. Vital Pregnancy Rate 2. Implantation Rate 3. Proportion of Subjects with Extreme Ovarian Responses, Defined as <4, ≥15 or ≥20 Oocytes Retrieved 4. Proportion of Subjects with Early OHSS (Ovarian Hyperstimulation Syndrome) and/or Preventive Interventions for Early OHSS 5. Proportion of Subjects with Cycle Cancellation Due to Poor Ovarian Response or Excessive Ovarian Response 6. Number of Oocytes Retrieved 7. Proportion of Subjects With <4, 4-7, 8-14, 15-19 and ≥20 Oocytes Retrieved 8. Percentage of Metaphase II Oocytes (Oocytes Inseminated Using ICSI [Intracytoplasmic Sperm Injection]) 9. Fertilisation Rate 10. Number and Quality of Embryos on Day 3 11. Number and Quality of Blastocysts on Day 5 12. Total Gonadotropin Dose 13. Number of Stimulation Days 14. Proportion of Subjects with Investigator-requested Gonadotropin Dose Adjustments 15. Frequency of Injection Site Reactions (Redness, Pain, Itching, Swelling and Bruising) Assessed by the Subject During the Stimulation Period 16. Abdominal Discomfort Related to Controlled Ovarian Stimulation as Assessed by a Visual Analogue Scale (VAS) 17. Changes in Body Weight 18. Changes in Maximum Abdominal Circumference 19. Proportion of Subjects with Treatment-induced Anti-follicle-stimulating Hormone (FSH) Antibodies 20. Proportion of Subjects with Late OHSS 21. Technical Malfunctions of the Administration Pen |

1. Ishihara, et al. (2021)

| Details of Inclusion criteria | 1. Informed Consent Documents signed prior to any trial-related procedures. 2. In good physical and mental health. 3. Japanese females between the ages of 20 and 40 years. 4. Infertile women diagnosed with tubal infertility, unexplained infertility, endometriosis stage I/II (defined by the revised American Society for Reproductive Medicine (ASRM) classification) or with partners diagnosed with male factor infertility, eligible for in vitro fertilization (IVF) and/or intracytoplasmic sperm injection (ICSI) treatment using ejaculated sperm from male partner. 5. Infertility for at least 1 year before randomization (not applicable in case of tubal or severe male factor infertility). 6. The trial cycle will be the subject's first controlled ovarian stimulation cycle for IVF/ICSI. 7. Hysterosalpingography, hysteroscopy, saline infusion sonography or transvaginal ultrasound documenting a uterus consistent with expected normal function (e.g., no evidence of clinically interfering uterine fibroids defined as submucous or intramural fibroids larger than 3 cm in diameter, no polyps and no congenital structural abnormalities which are associated with a reduced chance of pregnancy) within 1 year prior to screening. This also includes women who have been diagnosed with any of the above medical conditions but have had them surgically corrected within 1 year prior to screening. 8. Transvaginal ultrasound documenting presence and adequate visualization of both ovaries, without evidence of significant abnormality (e.g., no endometrioma greater than 3 cm or enlarged ovaries which would contraindicate the use of gonadotropins) and fallopian tubes and surrounding tissue without evidence of significant abnormality (e.g., no hydrosalpinx) within 1 year prior to screening. Both ovaries must be accessible for oocyte retrieval. 9. Early follicular phase (cycle day 2-4) serum levels of follicle stimulating hormone (FSH) between 1 and 15 IU/L (results obtained within 3 months prior to screening). 10. Body mass index (BMI) between 17.5 and 32.0 kg/m^2^ (both inclusive) at screening. |
| --- | --- |
| Details of Exclusion criteria | 1. Known endometriosis stage III-IV (defined by the revised ASRM classification). 2. One or more follicles >10 mm (including cysts) observed on the transvaginal ultrasound prior to start of stimulation on stimulation day 1 (puncture of cysts prior randomization is allowed). 3. Known history of recurrent miscarriage (defined as three consecutive losses after ultrasound confirmation of pregnancy (excl. ectopic pregnancy) and before week 24 of pregnancy). 4. Known abnormal karyotype of subject or of her partner. In case the sperm production is severely impaired (concentration <1 million/mL), normal karyotype, including no Y chromosome microdeletion, must be documented. 5. Active arterial or venous thromboembolism or severe thrombophlebitis, or a history of these events. 6. Any known clinically significant systemic disease (e.g., insulin-dependent diabetes). 7. Any known endocrine or metabolic abnormalities (pituitary, adrenal, pancreas, liver or kidney) which can compromise participation in the trial with the exception of controlled thyroid function disease. 8. Known tumors of the ovary, breast, uterus, adrenal gland, pituitary or hypothalamus which would contraindicate the use of gonadotropins. |
| Details of secondary outcomes | 1. Clinical Pregnancy Rate 2. Positive Beta Unit of Human Chorionic Gonadotropin (Beta-hCG) Rate 3. Vital Pregnancy Rate 4. Implantation Rate 5. Proportion of Participants with Cycle Cancellation Due to Poor or Excessive Ovarian Response 6. Proportion of Participants with Blastocyst Transfer Cancellation Due to Excessive Ovarian Response / OHSS Risk 7. Proportion of Participants with <4, 4-7, 8-14, 15-19 and ≥20 Oocytes Retrieved 8. Proportion of Participants with Extreme Ovarian Responses (Defined as <4, ≥15 or ≥20 Oocytes Retrieved) in Risk Population 9. Proportion of Participants with Preventive Interventions for Early Ovarian Hyperstimulation Syndrome (OHSS) 10. Proportions of Participants with Early OHSS (Including OHSS of Moderate/Severe Grade) and/or Preventive Interventions for Early OHSS 11. Proportions of Participants with Late OHSS (Including OHSS of Moderate/Severe Grade) 12. Number of Follicles on Stimulation Day 6 13. Number of Follicles at End-of-stimulation 14. Size of Follicles on Stimulation Day 6 15. Size of Follicles at End-of-Stimulation 16. Fertilization Rate 17. Number and Quality of Embryos 18. Number and Quality of Blastocysts 19. Circulating Levels of Endocrine Parameters (Follicle-stimulating Hormone [FSH], Luteinising Hormone [LH]) on Stimulation Day 6 20. Circulating Levels of Endocrine Parameters (Follicle-stimulating Hormone [FSH], Luteinising Hormone [LH]) at End-of-stimulation 21. Circulating Levels of Endocrine Parameter (Estradiol) on Stimulation Day 6 22. Circulating Levels of Endocrine Parameter (Estradiol) at End-of-stimulation 23. Circulating Levels of Endocrine Parameter (Progesterone) on Stimulation Day 6 24. Circulating Levels of Endocrine Parameter (Progesterone) at End-of-stimulation 25. Circulating Levels of Endocrine Parameters (Inhibin A) on Stimulation Day 6 26. Circulating Levels of Endocrine Parameters (Inhibin A) at End-of-stimulation 27. Circulating Levels of Endocrine Parameters (Inhibin B) on Stimulation Day 6 28. Circulating Levels of Endocrine Parameters (Inhibin B) at End-of-stimulation 29. Number of Stimulation Days 30. Total Gonadotropin Dose of FE 999049 31. Total Gonadotropin Dose of FOLLISTIM 32. Number of Participants With Adverse Events (AEs) Stratified by Intensity 33. Proportion of Participants Who Had Markedly Abnormal Value Changes From Baseline in Clinical Chemistry Parameters at End-of-stimulation 34. Proportion of Participants Who Had Markedly Abnormal Value Changes From Baseline in Hematology Parameters at End-of-stimulation 35. Proportion of Participants Who Had Markedly Abnormal Value Changes From Baseline in Clinical Chemistry Parameters at End-of-trial 36. Proportion of Participants Who Had Markedly Abnormal Value Changes From Baseline in Hematology Parameters at End-of-trial 37. Frequency and Intensity of Injection Site Reactions 38. Technical Malfunctions of the Administration Pens |

1. Qiao, et al. (2021)

| Details of Inclusion criteria | 1. Informed consent documents signed prior to screening evaluations. 2. In good physical and mental health in the judgement of the investigator. 3. Asian pre-menopausal females between the ages of 20 and 40 years. The subjects must be at least 20 years (including the 20th birthday) when they sign the informed consent and no more than 40 years (up to the day before the 41st birthday) at the time of randomization. 4. Infertile women diagnosed with tubal infertility, unexplained infertility, endometriosis stage I/II (defined by the revised American Society for Reproductive Medicine [ASRM] classification, 1996) or with partners diagnosed with male factor infertility, eligible for IVF and/or ICSI using fresh or frozen ejaculated sperm from male partner or sperm donor. 5. Infertility for at least 1 year before randomization for subjects <35 years or for at least 6 months for subjects 35 years (not applicable in case of tubal or severe male factor infertility). 6. The trial cycle will be the subject’s first controlled ovarian stimulation cycle for IVF/ICSI. 7. Regular menstrual cycles of 24–35 days (both inclusive), presumed to be ovulatory. 8. Hysterosalpingography, hysteroscopy, saline infusion sonography or transvaginal ultrasound documenting a uterus consistent with expected normal function (e.g., no evidence of clinically interfering uterine fibroids defined as submucous or intramural fibroids larger than 3 cm in diameter, no polyps and no congenital structural abnormalities which are associated with a reduced chance of pregnancy) within 1 year prior to randomization. 9. Transvaginal ultrasound documenting presence and adequate visualization of both ovaries, without evidence of significant abnormality (e.g., enlarged ovaries which would contraindicate the use of gonadotropins) and normal adnexa (e.g., no hydrosalpinx) within 1 year prior to randomization. Both ovaries must be accessible for oocyte retrieval. 10. Early follicular phase (cycle days 2-4) serum levels of FSH between 1 and 15 IU/l (results obtained within 3 months prior to randomization). 11. Negative serum Hepatitis B Surface Antigen (HBsAg), Hepatitis C Virus (HCV) and Human Immunodeficiency Virus (HIV) antibody tests within 2 years prior to randomization. 12. BMI between 17.5 and 32.0 kg/m^2^ (both inclusive) at screening. 13. Willing to accept transfer of 1–2 embryos. |
| --- | --- |
| Details of Exclusion criteria | 1. Known endometriosis stage III–IV (defined by the revised ASRM classification, 1996). 2. One or more follicles 10 mm (including cysts) observed on the trans vaginal ultrasound prior to randomization on stimulation day 1 (puncture of cysts is allowed prior to randomization). 3. Known history of recurrent miscarriage (defined as 3 consecutive losses after ultrasound confirmation of pregnancy (excl. ectopic pregnancy) and before week 24 of pregnancy). 4. Known abnormal karyotype of subject or of her partner/ sperm donor, as applicable, depending on source of sperm used for insemination in this trial. 5. Any known clinically significant systemic disease (e.g. insulin-dependent diabetes). 6. Known inherited or acquired thrombophilia disease. 7. Active arterial or venous thromboembolism or severe thrombophlebitis, or a history of these events. 8. Known porphyria. 9. Any known endocrine or metabolic abnormalities (pituitary, adrenal, pancreas, liver or kidney) with the exception of controlled thyroid function disease. 10. Known presence of anti-FSH antibodies (based on the information available in the subject’s medical records; i.e. not based on the anti-FSH antibody analyses conducted in the trial). 11. Known tumors of the ovary, breast, uterus, adrenal gland, pituitary or hypothalamus which would contraindicate the use of gonadotropins. 12. Known moderate or severe impairment of renal or hepatic function. 13. Any abnormal finding of clinical chemistry, hematology or vital signs at screening which is clinically significant as judged by the investigator. 14. Currently breast-feeding. 15. Undiagnosed vaginal bleeding. 16. Known abnormal cervical cytology of clinical significance observed within 3 years prior to randomization (unless the clinical significance has been resolved). 17. Findings at the gynecological examination at screening which preclude gonadotropin stimulation or are associated with a reduced chance of pregnancy, e.g., congenital uterine abnormalities or retained intrauterine device. 18. Pregnancy (negative urinary pregnancy tests must be documented at screening and prior to randomization) or contraindication to pregnancy. 19. Known current active pelvic inflammatory disease. 20. Use of fertility modifiers during the last menstrual cycle before randomization, including dehydroepiandrosterone (DHEA), metformin or cycle programming with oral contraceptives, progestogen or estrogen preparations. 21. Use of hormonal preparations (except for thyroid medication) during the last menstrual cycle before randomization. 22. Known history of chemotherapy (except for gestational conditions) or radiotherapy. 23. Current or past (1 year prior to randomization) abuse of alcohol or drugs. 24. Current (last month) intake of more than 14 units of alcohol per week. 25. Current or past (3 months prior to randomization) smoking habit of more than 10 cigarettes per day. 26. Hypersensitivity to any active ingredient or excipients in the medicinal products used in the trial. 27. Previous participation in the trial. 28. Use of any non-registered investigational drugs during the last 3 months prior to randomization. |
| Details of secondary outcomes | 1. Positive Beta Unit of Human Chorionic Gonadotropin (βhCG) Rate 2. Clinical Pregnancy Rate 3. Vital Pregnancy Rate 4. Implantation Rate 5. Ongoing Implantation Rate 6. Proportion of Subjects with Extreme Ovarian Responses 7. Proportion of Subjects with Early OHSS (Including OHSS of Moderate/Severe Grade) and/or Preventive Interventions for Early OHSS 8. Proportion of Subjects with Cycle Cancellation Due to Poor or Excessive Ovarian Response or Embryo Transfer Cancellation Due to Excessive Ovarian Response / OHSS Risk 9. Number of Follicles on Stimulation Day 6 10. Number of Follicles at End-of-stimulation (up to 20 Stimulation Days) 11. Size of Follicles on Stimulation Day 6 12. Size of Follicles at End-of-stimulation (up to 20 Stimulation Days) 13. Number of Oocytes Retrieved 14. Proportion of Subjects With <4, 4-7, 8-14, 15-19 and ≥20 Oocytes Retrieved 15. Percentage of Metaphase II (MII) Oocytes 16. Fertilization Rate 17. Number and Quality of Embryos 18. Circulating Concentrations of Luteinizing Hormone (LH) [ Time Frame: On stimulation Day 6 ] 19. Circulating Concentrations of LH [ Time Frame: End-of-stimulation (up to 20 stimulation days) ] 20. Circulating Concentrations of Estradiol [ Time Frame: On stimulation Day 6 ] 21. Circulating Concentrations of Estradiol [ Time Frame: End-of-stimulation (up to 20 stimulation days) ] 22. Circulating Concentrations of Progesterone [ Time Frame: On stimulation Day 6 ] 23. Circulating Concentrations of Progesterone [ Time Frame: End-of-stimulation (up to 20 stimulation days) ] 24. Circulating Concentrations of Inhibin A [ Time Frame: On stimulation Day 6 ] 25. Circulating Concentrations of Inhibin A [ Time Frame: End-of-stimulation (up to 20 stimulation days) ] 26. Circulating Concentrations of Inhibin B [ Time Frame: On stimulation Day 6 ] 27. Circulating Concentrations of Inhibin B [ Time Frame: End-of-stimulation (up to 20 stimulation days) ] 28. Circulating Concentrations of Follicle-stimulating Hormone (FSH) [ Time Frame: On stimulation Day 6 ] 29. Circulating Concentrations of FSH [ Time Frame: End-of-stimulation (up to 20 stimulation days) ] 30. Circulating Concentrations of FSH [ Time Frame: At oocyte retrieval ] 31. Total Gonadotropin Dose 32. Proportion of Subjects with Investigator-requested Gonadotropin Dose Adjustments 33. Number of Stimulation Days 34. Number of Participants with Adverse Events 35. Intensity of Adverse Events 36. Changes From Baseline in Clinical Chemistry Parameters: Alanine Aminotransferase, Alkaline Phosphatase, Aspartate Aminotransferase and Gamma Glutamyl Transferase 37. Change From Baseline in Clinical Chemistry Parameters: Bicarbonate, Blood Urea Nitrogen, Calcium, Chloride, Cholesterol, Glucose, Phosphate, Potassium and Sodium 38. Change From Baseline in Clinical Chemistry Parameters: Albumin and Protein 39. Change From Baseline in Clinical Chemistry Parameter: Lactate Dehydrogenase 40. Change From Baseline in Clinical Chemistry Parameter: Direct Bilirubin, Bilirubin, Creatinine, Urate 41. Proportion of Subjects with Markedly Abnormal Changes of Clinical Chemistry: Alanine Aminotransferase, Aspartate Aminotransferase, Bicarbonate, Calcium, Phosphate 42. Change From Baseline in Haematology Parameter: Erythrocytes 43. Change From Baseline in Haematology Parameters: Leukocytes and Platelets 44. Change From Baseline in Haematology Parameter: Haemoglobin 45. Change From Baseline in Haematology Parameter: Haematocrit 46. Change From Baseline in Haematology Parameter: Erythrocyte Mean Corpuscular Volume 47. Change From Baseline in Haematology Parameter: Erythrocyte Mean Corpuscular Haemoglobin 48. Change From Baseline in Haematology Parameter: Erythrocyte Mean Corpuscular Haemoglobin Concentration 49. Change From Baseline in Haematology Parameters: Basophils/Leukocytes, Eosinophils/Leukocytes, Lymphocytes/Leukocytes, Monocytes/Leukocytes and Neutrophils/Leukocytes 50. Proportion of Subjects with Markedly Abnormal Changes of Haematology Parameters: Leukocytes, Lymphocytes/Leukocytes 51. Number of Immune-related Adverse Events 52. Frequency of Injection Site Reactions 53. Intensity of Injection Site Reactions 54. Proportion of Subjects with Treatment-induced Anti-FSH Antibodies, Overall as Well as With Neutralizing Capacity 55. Intensity of Immune-related Adverse Events 56. Proportion of Subjects with Cycle Cancellations Due to an Adverse Event, Including Immune-related Adverse Events, or Due to Technical Malfunctions of the Administration Pen 57. Proportion of Subjects with Late OHSS 58. Proportion of Participants with Multi-fetal Gestation 59. Proportion of Participants with Early Pregnancy Losses 60. Proportion of Participants with Technical Malfunctions of the Administration Pen |
